# Supplementary material for: Integrated Methylome and Transcriptome Analysis Provides Insights into the DNA Methylation Underlying the Mechanism of Cytoplasmic Male Sterility in Kenaf (Hibiscus cannabinus L.)
Source: Int J Mol Sci. 2022 Jun 20;23(12):6864. doi: 10.3390/ijms23126864 (PMC9224340; doi:10.3390/ijms23126864)
Supplement: Supplementary file 1 [file ijms-23-06864-s001.zip › Table S7 Primer sequences for qRT-PCR.pdf]

Table S7 Primer sequences for qRT-PCR.

Gene name	Sequence (5′-3′)	
	Forward	Reverse
His3（*reference gene*）	ATGGCCCGTACCAAGCAAACC	TAAGCACGCTCGCCCCTGAT
atp8	CATTGAGGTTGGCGAGATAATAAAG	AAGTGACAGTCGTTTCCCATCG
beta-fructofuranosidase	CACACATACGACAAGAGCGGC	AAGAGGGTCAGAGTGGTCAGCAG
beta-glucosidase	CGGGATTCCTTATGTCTTTGCTC	CTTCGTTGATGCCCCTATTTGTC
COX2	AGCAGCAAAGATTGAAGTGAAAGAG	AGAGTAACAAAGTTGAACAGACAGCC
DDM1	GCACTGCTACGGAACGAAGAAAC	CACAAAAGAGGTAAAACTCCGCATC
DME	ACTGGAAAAGCAAGATGGTGAAGG	GCAAAGACACTATGGATTCCCTGG
DRM1	GACGAAAATGGGGTATTCAGAGGC	CAGAATGGCTTCCTATCTTCAACCG
DRM2	ATGTCCCTCTCTGGTTGGTTTGTTC	TGGTAACTCGTTTCAGGTTGACACAG
fatty acid amide hydrolase	CTTCCGACGCTGGACGATAG	ATAAACTGCTCCGCAACCCTC
GA2ox6	GGCTCAATCACTATCCACCATCC	GACAGGGACCCAGACAGCATC
IAA32	GGATGGAGTTTTGGTTGGTCGC	ATACGCAGCCGTTTCACCCG
ILR1	ACCCTCCAACCATAAACGATAACG	TTTGGGCTGCTCTCATCTTGC
JMJ705	CAGGGTTGAGGGCTATTGTGG	CCTGAATGATAGGCTCTCGGG
MADS-box protein AGL29	TCGCTACCCTTTGTGCCGTTC	GATTTAGAAACCGCTCCGTCACTG
MADS-box protein AGL30	GCAGAAAAGGGATGCTACCACTC	TACCAGTTTTGCTTGGCGATG
MADS-box protein AGL62	ATTAGAGGATAAGAGGAAGCGTGGC	GATTTGAGTTGCTTCAGTGTGGAGAG
MADS-box protein AGL66	CGTGGTGCGGAATAAAGAGTT	GTTTGAGTTAGTGGTGGCGGT
MADS-box transcription factor	TTTTGGAGGAGGATTACAAGCACC	GCATCTGAGAGTTGTAGTCCTGGG
MADS-box transcription factor 23	TTTGAACCAGAATCATCTACCATAAGG	GCCTGTCCAACTCCAACTAAGCC
MADS-box transcription factor 27	TGCGATGCCGAGGTTGG	TACTTGTTGCCTCAAACTTGCTACC
MET1	TGGACTGCTGTTTTTGGTTTCACTG	GTTCAGTTCCGTTAGCCACCTCAG
MYB21	TTGACTACCGTTTCTGATTTCATTTC	CGTCCCTGAACTGTTTGCTGTC
MYB26	CAGTGCCCAAACTCTGTGAAATCC	GCAGGAGCAGCAGGCATCG
phytochrome B	AATCACCGCCTACTTGTCTAAAATCC	GTAGGCGATGATACGGAAACTGG
protein WLIM2b	AAATCCGAAAAGCAAAAACAACTG	CCTCCACTGTCACCTTCTCTAACG
pyruvate kinase	TAAAGGGTGATGAGAAACTGATAAGC	GTCGCAAGACAAGACGGTGAAC
ROS1	ACTACACCACAGCCAAAACAAAGG	GGAGGATTGATGCGTCTTTTTCTAC
